# What do we know about extracellular vesicles in patients with idiopathic pulmonary fibrosis? a scoping review

**DOI:** 10.3389/fimmu.2025.1541645

**Published:** 2025-07-02

**Authors:** Maria Conti, Alvise Casara, Graziella Turato, Simonetta Baraldo, Mariaenrica Tinè, Umberto Semenzato, Elisabetta Cocconcelli, Davide Biondini, Marco Damin, Marina Saetta, Manuel G. Cosio, Elisabetta Balestro, Paolo Spagnolo, Erica Bazzan, Nicol Bernardinello

**Affiliations:** ^1^ Department of Cardiac, Thoracic, Vascular Sciences and Public Health, University of Padova and Padova City Hospital, Padova, Italy; ^2^ Centro Cardiologico Monzino IRCCS, Milan, Italy; ^3^ Department of Medicine, University of Padova, Padova, Italy; ^4^ Meakins-Christie Laboratories, Respiratory Division, McGill University, Montreal, QC, Canada

**Keywords:** extracellular vesicles, interstitial lung diseases, MISEV, idiopathic pulmonary fibrosis, lung fibrosis

## Abstract

Extracellular Vesicles (EVs), released by all cell types and detectable in biological samples, carry a variety of biological molecules. These molecules mediate communication and signaling with both local and distant cells, potentially playing a role in the pathogenesis of diseases, including Interstitial Lung Diseases and, more specifically, Idiopathic Pulmonary Fibrosis. To better understand the role of EVs in IPF, a systematic search was performed in PubMed, Scopus, and Ovid databases. These searches were conducted from January 1^st^, 2019, the period during which the MISEV 2018 guidelines were published, to August 31^st^, 2024. The SANRA scale was used for quality assessment. A total of 691 papers were screened, and 16, in the end, were definitively enrolled for our evaluation. The studies were reviewed in the following steps: 1) the nomenclature used to define EVs; 2) conformity with the MISEV 2018 guidelines; 3) the biological samples used to isolate EVs; 4) the main conclusion of each manuscript. There was significant heterogeneity among the publications in all the aforementioned steps, such as the type and source of EVs and the analysis of EVs content, primarily a wide array of different miRNAs in the various publications. Despite these differences, the emerging role of EVs and their potential usefulness both in therapies and clinical practice is of growing interest.

## 1 Introduction

Interstitial lung diseases (ILDs) represent a broad spectrum of lung disorders, mainly characterized by inflammation and progressive fibrotic remodeling of lung interstitium, leading to a progressive impairment of ventilation and gas exchange that gives rise to breathlessness and, in many cases, respiratory failure and death (1). More than 200 different conditions, ranging from very rare to more common diseases such as Connective Tissue diseases-ILDs, Sarcoidosis, Hypersensitivity Pneumonitis, and Idiopathic Pulmonary Fibrosis (IPF), are grouped in the term “interstitial lung disease”. However, Idiopathic Pulmonary Fibrosis represents still today the most frequent and the most terrible form in the group of idiopathic. The hallmark of IPF is the usual interstitial pneumonia (UIP) pattern, which can be present both in histological specimens and radiological imaging. Antifibrotic therapies, Nintedanib and Pirfenidone, are now presently available for Idiopathic Pulmonary Fibrosis (IPF) treatment, and they showed efficacy in improving lung function and survival, although prognosis remains extremely poor (2, 3). The severe morbidity and poor prognosis of this disease and the many unmet clinical needs deserve the development of new diagnostic and therapeutic approaches that could improve the management of these patients.

Extracellular Vesicles (EVs) are lipid bilayer-enclosed spheres that cannot replicate on their own (i.e., do not contain a functional nucleus), released by all cell types and detectable in biological samples (BAL sputum, blood, and plasma). EVs can be used to study several diseases (including lung diseases) and could become an important clinical tool that might forward the understanding of the mechanisms and even therapies of the ILDs. As indicated in the MISEV guidelines (4–6), in particular in MISEV 2018 guidelines, subtypes of EVs can be identified based on physical characteristics (such as size and density), biochemical composition (surface marker expression), or descriptions of conditions or cell of origin (podocyte EVs, hypoxic EVs, large oncosomes, apoptotic bodies) (5).

Among EVs, exosomes are small extracellular vesicles ranging from 30 to 200 nm from internal compartments of the cell that are released via the multivesicular body (MVB). Conversely, microvesicles are categorized as medium-sized extracellular vesicles, measuring between 100 and 1000 nm, which form through direct budding from the plasma membrane, thereby leading to their release into the extracellular space. In contrast, apoptotic bodies represent larger extracellular vesicles exceeding 1000 nm in size and are generated through membrane blebbing during the disassembly of apoptotic cells (6).

The MISEV guidelines (Minimal Information for Studies of Extracellular Vesicles), first introduced by the International Society for Extracellular Vesicles (ISEV) in 2014 and updated in 2018 and 2023, were developed to improve the rigor, reproducibility, and comparability of research involving extracellular vesicles (EVs). These guidelines provide comprehensive recommendations on terminology, isolation, characterization, and functional studies of EVs, and have become the international standard for researchers in the field.

Both the MISEV 2018 and the updated MISEV 2023 guidelines recommend using the umbrella term “extracellular vesicles” (EVs) to refer to vesicles of all sizes, regardless of their presumed biogenesis. This recommendation stems from the difficulty in conclusively determining the cellular origin and mechanism of release of EV subtypes (such as exosomes or microvesicles), due to the lack of definitive molecular markers and the overlapping size ranges and compositions of these particles.

Although there is no strict consensus on the exact size boundaries, vesicles smaller than 200 nm are typically referred to as “small EVs”, while those larger than 200 nm are termed “large EVs”, based on their diameter after isolation. As a result, developing standardized methods capable of selectively isolating and characterizing specific EV subpopulations remains a major challenge in the field.

EVs can carry a variety of biological molecules (RNA, miRNAs, cytokines, lipids, etc.) that mediate the communication and signaling with local and distant cells (7), and their investigation might potentially reveal the different cellular pathways and pathophysiological processes involved in the pathogenesis of the disease being evaluated (1).

The study of EVs has become an important tool in investigating possible new diagnostic and therapeutic approaches to Interstitial Lung Diseases. However, the interpretation of these publications might be limited due to the high heterogeneity in the terminology, processing, and quantification of EVs. This scoping review aimed to identify the evidence reported in the recent literature, forwarding the understanding of the utility of EVs in patients with IPF.

## 2 Methods

Bibliographic searches were performed using PubMed, Scopus, and Ovid databases. The search time was restricted from 1.01.2019 [when the MISEV 2018 guidelines were published (5)] to 31.08.2024. The specific search strategy was: “Extracellular Vesicles” AND [(Diffuse parenchymal lung disease) OR (Interstitial lung diseases) OR (Idiopathic Pulmonary Fibrosis) OR (Interstitial pneumonia) OR (Interstitial Pneumonitis) OR (Lung Fibrosis) OR (Respiratory Interstitial Diseases) OR (Idiopathic Pneumonitis) OR (Idiopathic Interstitial Pneumonia)]. We decided to use the specificity of the term “Extracellular Vesicles” to restrict our research according to the definition proposed in the latest MISEV guidelines (5). Moreover, we added multiple terms related to IPF to capture all relevant studies in the literature. We also reviewed references cited in selected articles that matched the aim of this scoping review and read the full texts.

**Figure 1:**
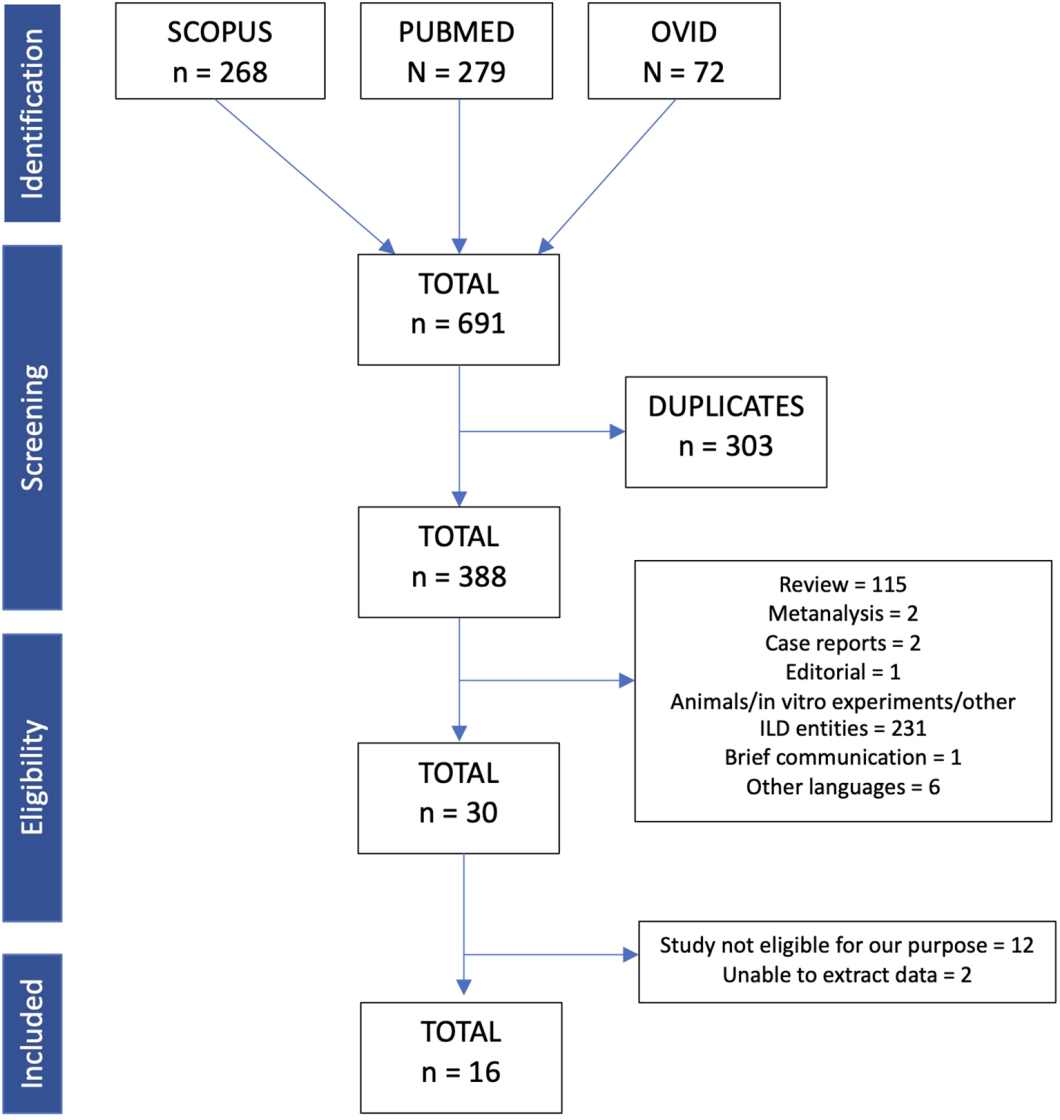
Flow chart of included articles for this scoping review.

Inclusion criteria were: I) studies involving IPF patients; II) studies published between January 2019 and August 2024; III) original articles. Exclusion criteria were: I) animal/*in vitro* experiments; II) case reports; III) reviews; IV) papers published in languages other than English; V) conference abstracts; and VI) systematic reviews or meta-analyses. We considered only studies available in full-text form. The literature retrieved from the database search was imported into the Rayyan platform for deduplication. All duplicate records, i.e., papers retrieved from multiple databases, were identified and removed before the screening process. **Figure 1** presents the flow diagram of the study design.

Two investigators (M.C. and A.C.) independently reviewed the identified manuscripts to determine their eligibility for inclusion in the review. This scoping review was conducted in accordance with the Preferred Reporting Items for Systematic Reviews and Meta-Analyses extension for Scoping Reviews (PRISMA-ScR) checklist (8). In the supplemental material, we reported the score used for the quality assessment of selected studies (SANRA score) (9).

## 3 Results

### 3.1 Literature overview of extracellular vesicles and idiopathic pulmonary fibrosis

The search retrieved a total of 691 articles. Of the 691 articles, 303 were eliminated because of duplicates. To ensure a correct elimination, the platform Rayyan was used. Out of the 388 articles, 115 were excluded because of review. Others were also excluded because they were not in line with our purpose (the majority of them treated other lung diseases such as hypersensitivity pneumonitis or sarcoidosis or were involved *in vitro* experiments/animal models).

In the end, only a minority of original papers (n= 30) investigated EVs directly isolated from human biological samples from patients with IPF. After a deep investigation, 16 were finally considered for our scoping review (10–25). In line with the aim of this review, the following steps taken in the definition and acquisition of EVs in these manuscripts were analyzed for:

1. the nomenclature used to define EVs;
2. the conformity with the MISEV 2018 guidelines for the isolation methods;
3. the biological samples used to isolate EVs and the cellular origin for EVs;
4. main conclusion of each manuscript.

All the manuscripts included in the analysis are reported in **Tables 1**, **2**.

**Table 1:** Clinical characteristics.

Reference	IPF patients enrolled	Specimens	Age (years ± SD, range or median)	Males (%)	Subjects	Baseline FVC (%)	Smoke habits (%)
Neri T. 2019 (10)	10	Plasma	Not available	Not available	ILD patients (IPF, Sjogren, possible UIP, NSIP, asbestosis) vs HS	Not available	Not available
Wan X. 2020 (11)	30	Lung tissue	62.13 ± 7.66	Not available	IPF vs. HS	Not available	Not available
Guiot J. 2020 (12)	33	Sputum and plasma	Cohort 1: 68 ± 14 Cohort 2: 71 ± 9	Cohort 1: 68 Cohort 2: 86	IPF vs. HS	Cohort 1: 74 ± 19 Cohort 2: 77 ± 16	Cohort 1: 79 Cohort 2: 93
Kadota T. 2020 (13)	20	Lung tissue/lung fibroblast	71.4 ± 6.0	80	IPF vs. HS	90.63 ± 15.8	P/Y 66.8 ± 65.8
Lacedonia D. 2021 (14)	61	Serum	68.03 ± 6.33	86	IPF vs. HS	70.83 ± 16.00	Not available
Merino A. 2021 (15)	6	MSCs from adipose tissue of HDs	Range: 64 - 77	Not available	IPF	Short telomere group: 78.3 ± 4.3 Normal telomere group: 71 ± 4.9	Not available
Kaur G. 2021 (16)	16	BALF, Lung Tissue	Bal cohort: 76.5 ± 11.4 Lung Tissue cohort: 68.9 ± 9.6	Bal cohort: 75 Lung Tissue cohort: 62	IPF vs. COPD/Emphysema vs. HS	Not available	Bal cohort: 50 Lung Tissue cohort: 75
D’alessandro M. 2021 (17)	90	Serum	71 (IQR 66-75) years	77	IPF vs. HS	68 (IQR 59–91)	53
Sato S. 2021 (18)	Derivation cohort: 7 Validation cohort: 16	BALF	Derivation cohort: 59.4 ± 4.6 Validation cohort: 71.1 ± 3.8	Derivation cohort: 57 Validation cohort: 44	IPF (UIP or fNSIP pattern)	Derivation cohort: 85.6 ± 5.9 Validation cohort: 82.5 ± 3.7	Derivation cohort: 57.1 Validation cohort: 43.8
Shaba E. 2021 (19)	3	BALF	69 ± 5	100	IPF	50.3 (range 39 - 62)	67
Elliot S. 2022 (20)	16	Urine, Lung tissue, serum	55-79	100	IPF vs. HS	Not available	Not available
Hayek H. 2024 (21)	13	Lung tissue, plasma	62.76 ± 6 2.41	77	IPF vs. HS	41 ± 4.57	Not available
D’alessandro M. 2023 (22)	Study cohort: 17 Validation cohort: 44	BALF	Study cohort: 64.7 ± 23.8 Validation cohort: 62.6 ± 19.8	Study cohort: 76 Validation cohort: 70	IPF vs. Sarcoidosis vs. HP	Study cohort: 81.9 ± 27.5 Validation cohort: 79.6 ± 10.4	Study cohort: 65 Validation cohort: 34
Asghar S. 2023 (23)	8	Lung tissue/Human bronchial epithelial cells	65.6 ± 7.2	75	IPF vs. HS	81.5 ± 20.15	50
Fujita Y. 2023 (24)	20	Lung tissue/lung fibroblast	71.4 ± 6.0	80	IPF	90.63 ± 15.8	P/Y 66.8 ± 65.8
Tomoto M. 2024 (25)	206	Serum	72.8 ± 9.33	74.8	IPF vs. HS	Not available	Not available

IPF, Idiopathic pulmonary fibrosis; FVC, Forced vital capacity; EVs, Extracellular vesicles; ILD, Interstitial lung disease; UIP, Usual interstitial pneumonia; NSIP, Nonspecific interstitial pneumonia; HS, Healthy subjects; P/Y, packyears; HDs, Healthy donors; BALF, Bronchoalveolar lavage fluid; COPD, Chronic obstructive pulmonary disease; HP, Hypersensitivity pneumonitis.

**Table 2:** EVs isolation and assessment.

Reference	Specimens	EVs nomenclature	EVs isolation	EVs quantification and characterization methods	Biomolecules	miRNAs
Neri T. 2019 (10)	Plasma	Extracellular vesicles	/	Flow cytometry	Endothelial markers: CD31 and CD62E	/
Wan X. 2020 (11)	Bone marrow mesenchymal stem cells	Extracellular vesicles	Ultracentrifugation	BCA Protein assay + TEM + NTA + WB	Exosomes markers: CD63 and CD81	miRNA-29b-3p
Guiot J. 2020 (12)	Sputum and plasma	Exosomes	Ultracentrifugation	BCA Protein Assay + NTA + WB	Exosomes markers: CD9, CD63 and CD81	miR-142-3p, miR-200c-5p, Let-7d-5p, miR-33a-5p
Kadota T. 2020 (13)	Lung tissue/lung fibroblast	Extracellular vesicles	Ultracentrifugation	Quant-iT Protein Assay + NTA + TEM	/	miR-23b-3p; miR-494–3p; miR-145-5p
Lacedonia D. 2021 (14)	Serum	Exosomes	Ultracentrifugation	Spectroscopy + WB	Exosomes markers: CD81	miR‐16; miR‐21; miR‐26a; miR‐210; miRLet‐7d
Merino A. 2021 (15)	MSCs from adipose tissue of HDs	Membrane Particles artificially generated	Generation: Extrusion process	Cryo-TEM + NTA + nLC-MS/MS	Matrix metalloproteinases by MMP Activity Assay kit	/
Kaur G. 2021 (16)	BALF, Lung Tissue	Extracellular vesicles	Plasma/serum exosome isolation kit (for BALF exosomes) or Ultracentrifugation (for lung tissue exosomes)	NTA + TEM + Immunoblot	Exosomes markers: CD9, CD63 and CD81	Multiple analysis (including: miR-122-5p)
D’alessandro M. 2021 (17)	Serum	Extracellular vesicles	Ultracentrifugation	WB + Bradford assay + MACSPlex Exosome Kit	37 surface epitopes (including CD9, CD63, CD81)	/
Sato S. 2021 (18)	BALF	Extracellular vesicles	Total Exosome Isolation kit + Ultracentrifugation	EM + BCA Protein Assay + Flow cytometry	Exosomes markers: CD9 and CD63	miR-21-5p
Shaba E. 2021 (19)	BALF	Extracellular vesicles	Ultracentrifugation	TEM + 2DE + LC-MS/MS	Complete analysis of protein content	/
Elliot S. 2022 (20)	Urine, Lung tissue, serum	Exosomes	Ultracentrifugation	MACSPlex surface protein analysis + TEM + WB	Exosomes markers: CD1, CDC9, CD9, CD63 and CD81	miR-let-7d, miR-29a-5p, miR-181b-3p and miR-199a-3p
Hayek H. 2024 (21)	Lung tissue, plasma	Exosomes	Ultracentrifugation	NTA + RT-PCR + WB	Exosomes markers: CD63 and CD81	miR-143-5p and miR-342-5p
D’alessandro M. 2023 (22)	BALF	Exosomes	/	MACSPlex Exosome kit	37 surface epitopes (including CD9, CD63, CD81)	/
Asghar S. 2023 (23)	Lung tissue/Human bronchial epithelial cells	Extracellular vesicles	Ultracentrifugation or Exoquick	NTA + TEM + WB + ExoELISA	Multiple analysis (including CD9, CD63 and CD81)	Multiple analysis (including: miR-411-5p, miR-7-5p, miR137-3p, miR195-5p)
Fujita Y. 2023 (24)	Lung tissue/lung fibroblast	Extracellular vesicles	Ultracentrifugation	Quant-iT Protein Assay + NTA+ Cryo-TEM	Exosomes markers: CD9, CD63 and Caveolin-1	miR-19a
Tomoto M. 2024 (25)	Serum	Extracellular vesicles	MagCapture isolation kit (phosphatidylserine-positive EVs)	Bayesian network and edgeR of a proteome dataset	Proteome analysis	/

EVs, Extracellular vesicles; NTA, Nanoparticle Tracking Analysis; CD, Cluster of differentiation; WB, Western blot; TEM, Transmission Electron Microscopy; BCA, Bicinchoninic acid assay; MSCs, Mesenchymal stromal cells; nLC-MS/MS, nano-liquid chromatography mass spectrometry.

### 3.2 The nomenclature used to define EVs

The nomenclature of what was initially called “platelets cell debris” by Chargaff in 1946, and what we now call “Extracellular Vesicles (EVs)”, has been confusing because of a lack of standardization (membrane particles, exosomes, microvesicles, microparticles) (26). These differences made the comparison between the increasing numbers of scientific studies on EVs challenging due to the many definitions of the terms used, which led to the creation of specific guidelines defining the use of appropriate terms and methodologies to isolate and characterize EVs. The guidelines (5) endorse “Extracellular Vesicles (EVs)” as a generic term for particles naturally released from the cell that are delimited by a lipid bilayer and cannot replicate. Given the current lack of consensus regarding specific markers for EV subtypes, attributing an EV to a particular biogenesis pathway (such as exosomes or membrane-derived particles) remains challenging in the absence of direct observation through live imaging techniques. Authors are encouraged to establish reliable subcellular markers or use operational terms referring to physical characteristics (small, medium, and large), biochemical composition, or cell origin instead of historically ambiguous terms like “exosome” or “microvesicle (5)”. Therefore, we examined the terminology used in the 16 articles on EVs from patients with IPF published after the appearance of the guidelines. Among these articles, 10/16 used the term “extracellular vesicles”, 5/16 utilized the term “exosomes”, and 1/16 employed “membrane particles”. This adherence to outdated terminology could pose a misinterpretation of results and reduce accurate comparisons across studies, as emphasized in the recent MISEV 2023 Guidelines (6).

### 3.3 MISEV 2018 guidelines for the EVs characterization methods

Although the MISEV 2018 Guidelines provide clear definitions and standardized terminology in EVs research, the establishment of consistent methodologies for EVs isolation and characterization remains an ongoing challenge. The guidelines recommends that each preparation of EVs be:

1. defined by quantitative measures of the source of EVs (e.g. number of secreting cells, volume of biofluid, mass of tissue);
2. characterized to the extent possible to determine abundance of EVs (total particle number and/or protein or lipid content);
3. tested for presence of components associated with EV subtypes or EVs generically, depending on the specificity one wishes to achieve;
4. tested for the presence of non-vesicular, co-isolated components” (5, 6).

In light of these principles, we conducted a review of 16 articles focusing on EVs derived from IPF patients to assess whether the methods employed for their characterization aligned with the MISEV 2018 guidelines. Our analysis revealed that, although the term “extracellular vesicles”, was commonly used, not all studies applied at least two complementary methods for EVs characterization in accordance with the guidelines. The proper identification and naming of the EVs is thus a fundamental step for the research conclusions and further comparison among different studies.

Before the release of the MISEV 2018 guidelines, several studies had already explored the diagnostic, prognostic, and therapeutic potential of EVs in IPF. However, the lack of standardized terminology and methodological frameworks resulted in significant heterogeneity. Researchers used various terms such as “exosomes”, “microparticles”, or “vesicular particles” to describe EVs, and each group employed their own protocols for isolation and analysis. For example, Makiguchi et al. (27) demonstrated that serum EVs containing miR-21-5p were elevated in IPF patients and associated with increased mortality risk. Similarly, another study revealed that EVs from IPF patients and fibrotic models carried profibrotic mediators like WNT5A and actively contributed to lung fibrogenesis, emphasizing their diagnostic, prognostic, and pathogenic roles (28). Furthermore, Liu et al. (29) identified differential miRNA expression profiles in EVs from IPF patients, while Bacha et al. (30) observed higher levels of endothelial microparticles in patients with severe functional impairment. Additional research, including a multi-cohort investigation in 2018 and work by Shentu et al. (31), examined the involvement of mesenchymal EVs in fibrogenesis and their potential therapeutic applications. Collectively, these findings underscore the significant interest in EVs even before 2018. However, they also highlight the methodological variability that hindered comparability and reproducibility across studies.

### 3.4 Heterogeneity of biological samples

**Figure 2:**
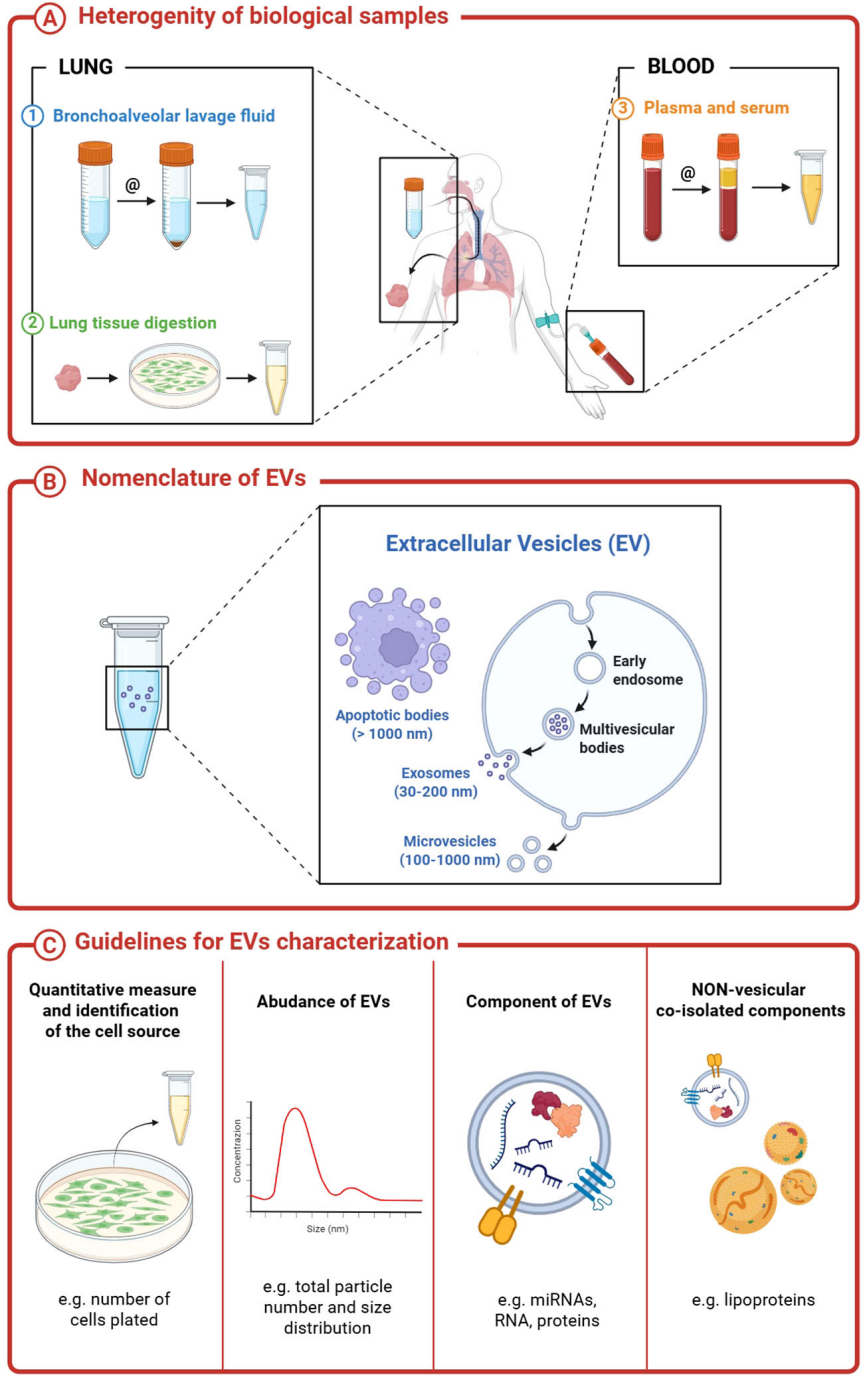
Extracellular vesicles nomenclature, sources and characterization.

EVs secreted by living cells carry active biomarkers that are released into the extracellular space, where they can be involved in various physiological and pathological processes. EVs can be isolated from any type of bodily fluid, such as blood, urine, or lung Bronchoalveolar Lavage (BAL), among others (**Figure 2**). Among the 16 studies on IPF (10–25) patient-derived EVs included in this review, 69% (11/16) utilized lung-derived biological samples for EV isolation. Specifically, 25% (4/16) used bronchoalveolar lavage (BAL) fluid, while 43% (7/16) obtained cells directly from lung tissue. Sputum was employed in 6% (1/16) of the studies. Additionally, plasma, serum, and urine were used in 3/16, 4/16, and 1/16 of the studies, respectively. Curiously, in one study, EVs were isolated from healthy kidney adipose tissue, aiming to investigate the antifibrotic role of EVs derived from human mesenchymal stromal cells (MSCs) from adipose tissue in an *in vitro* model of pulmonary fibrosis (15). An important consideration emerging from our analysis is that not all the manuscripts investigate EVs originating directly from the lungs (like bronchoalveolar lavage, lung tissue, and sputum) site of the disease being investigated, which ought to best reflect the underlying lung abnormalities. Moreover, the bronchoalveolar lavage (BAL) technique is crucial for obtaining representative specimens, as it is designed to reflect the composition of inflammatory cells and their secreted products within the distal airspaces (32). Bronchoscopy and thoracic surgery are invasive procedures and may account for the limited direct lung investigations in some studies despite their potential to provide valuable insights into the local microenvironment.

Given these difficulties, it is not surprising that blood specimens (entire blood, serum, or plasma) are often preferred because they are more accessible and less invasive than BAL performers with bronchoscopy or video-assisted thoracoscopic (VATS). It is crucial to recognize that EVs derived from BAL or lung tissue provide distinctly different information compared to blood-derived EVs; the latter may lack, obscure, or dilute lung-specific abnormalities due to the contribution of EVs from other organs and systems. In a study by Tinè et al. (33) comparing EVs derived from BAL and blood in smokers with and without COPD and non-smoking controls, none of the differences observed in BAL (amount of EVs derived from alveolar macrophages, endothelial and epithelial cells) were found in blood where the amount the different type of EVs was similar in the three groups of subjects examined, suggesting that BAL is a better media than blood to study EVs in lung diseases. Furthermore, there are important differences in the EVs population derived from whole blood, serum, and plasma, especially when miRNAs are studied. Before establishing protocols to investigate EVs in blood, serum, or plasma for the analysis of EVs in IPF, maybe a protocol comparing the findings from BAL with those from plasma would serve as a guide on how to proceed with the investigation. In this sense, the findings in BAL and lung tissue might guide what to look for in blood samples.

In addition, as highlighted in the MISEV 2018 guidelines, each biological fluid possesses distinct biophysical and biochemical properties that must be carefully considered when isolating EVs. For example, plasma and serum contain a variety of non-EV lipid-based structures—such as low-, very-low-, and high-density lipoproteins—that can be co-isolated with EVs to varying extents. To address this, the MISEV guidelines recommend the use of apolipoproteins A1, A2, and B (APOA1/2, APOB) and albumin (ALB) as the most reliable negative markers currently available to help distinguish EVs from contaminating non-vesicular components.

## 4 What was studied and what was learned from these investigations

Our comprehensive analysis of 16 studies demonstrates that EVs possess significant capacity to acquire and transport diverse molecular and protein cargoes, potentially influencing key stages of disease progression, underscoring the promising research opportunities that EV investigations offer across the spectrum of interstitial lung diseases (ILDs). In these papers, EVs were retrieved from variable sources of which lung cells (fibroblasts, epithelial cells, lymphocytes, macrophages alone, and often together with other cells) derived from tissue digestion, and BAL was the most frequently used. From papers in which EVs were obtained from the isolated lung cells, 3 papers investigated an array of miRNAs linked to fibrotic pathways such as collagen production, wound healing, and fibrotic invasion (10, 16, 21), 2 papers highlighted the role of miRNAs in epithelial senescence and mitochondrial damage processes (13, 23) and one paper focused on cMyc activation and cancer progression (24).

When BALF was used, 50% of the papers investigated miRNA. In one study, the authors analyzed and compared miRNA profiles in bronchoalveolar lavage fluid (BALF) and lung tissue-derived exosomes across distinct cohorts, including healthy non-smokers, smokers, and patients diagnosed with either chronic obstructive pulmonary disease (COPD) or idiopathic pulmonary fibrosis (IPF) (16). In patients with IPF, there was a high number (n=55) of differentially expressed miRNAs in the lung-tissue-derived exosomes not seen in non-smoking controls, which indicates that in a complex disease like IPF, a large number of miRNAs would be expected to be present. Three papers do not examine a specific pathway, only different markers’ expression on EVs, while one explores the contribution of extracellular matrix stiffness to fibrogenesis (18, 21). In addition, a recent proteomic study conducted on BALF-derived EVs from IPF patients provided a comprehensive characterization of their internal protein cargo, unveiling molecular pathways not previously associated with the disease—such as cytoskeletal remodeling, adenosine and adrenergic signaling, and lipid metabolism—thereby expanding our understanding of the multifaceted roles EVs may play in fibrogenesis beyond miRNA-mediated mechanisms (21).

Of the papers investigating serum or plasma samples (10, 12, 14, 16, 20, 21, 25), 60% of them analyzed miRNA in the context of 3 general fibrotic pathways: collagen deposition, alfa-SMA production, and fibroblast migration. As can be seen, the available studies are very heterogeneous in terms of EV source, patient sample characteristics, and the technology used for the separation of EVs.

It is well noted that IPF is an age-related disease (34), for this reason the molecular mechanism of senescence is deeply investigated. Intriguingly, Asghar and coworker demonstrated that small EVs isolated from IPF-diseased human bronchial epithelial cells transfer senescence to neighboring healthy cells, promoting the fibrotic stage (23). Similar results were also reported by Kadota and coworkers (13). More in detail, EVs from IPF fibroblast with elevated levels of microRNA-23b-3p (miR-23b-3p) and miR-494-3p epithelial-cell senescence. Of interest, the levels of miR-23b-3p and miR-494-3p correlated positively with the disease severity. Moreover, in another study, the authors demonstrated that EVs containing miR-143-5p and miR-342-5p seem to induce the profibrotic response in fibroblast (12).

As noted, miRNA are frequently investigated in EVs studies. However, only two were investigated in more than one paper in our research: miR-let7d and miR142-3p. The human lethal-7 (let-7) (35) microRNA family exerts a profound influence on epithelial-to-mesenchymal transition (EMT) and the formation of cancer-initiating cells, also playing a crucial role in regulating developmental processes and carcinogenesis. Let-7d is a small RNA with great power, but in different cell genetic backgrounds, it acts in different ways, which makes this molecule still mysterious. Let-7d is directly transcriptionally inhibited by the key profibrotic cytokine TGF-b. Finally, miR142-3p plays an important role in development and is significantly upregulated in the sputum and plasma of patients with IPF, where it might have an antifibrotic role. The selection of specific miRNAs for investigation in the context of fibrotic diseases or any pathological condition is complicated by the multitude of potential candidates available for study. In a recent review, Yang (36) presented a list of 60 different miRNAs expressed by exosomes and EVs in pulmonary fibrosis, some with a suppressor and some with an upregulation function. Not surprisingly, as often happens in the available studies, the choice of miRNAs to investigate is difficult. To complicate matters is the general belief that each miRNA could interact with more than 200 mRNA, and conversely, a particular mRNA could be regulated by multiple miRNAs. Another important consideration is the experimental setting for the source of miRNA since *in vitro* single-cell EV-miRNA, although specific, would not be a reflection of the enormous quantity of miRNA acting in a very complex disease like IPF where multiple cells mRNAs and proteins are at play. For instance, a recent study investigating urinary miRNA profiles in patients with bladder cancer identified over 2,000 distinct miRNA species (37). Despite significant advancements in EVs research, several critical limitations remain—chief among them is the current inability to reliably determine the cellular origin of EVs within complex biological fluids. This constraint hampers our understanding of their biological relevance and functional roles. One of the major unresolved questions is whether EVs derived from different cell types interact or influence one another within biofluids, potentially altering their molecular composition and confounding downstream analyses. Furthermore, the mere presence of EVs does not inherently imply biological activity or functional significance, especially in the absence of definitive data regarding their source. To overcome these challenges, future research should focus on the development of innovative strategies to trace the origin of EVs—such as molecular barcoding, lineage-specific markers, or cell-type-selective isolation techniques—and to identify functionally distinct EV subpopulations with greater precision.

Another consideration regards the content of the small (<150μm) -so-called exosomes- and the medium-large EVs. If small EVs are generated from Multi Vesicular Bodies (MVB) while larger EVs are membrane-derived, it is very likely their content, mRNA and/or other proteins- will be different. Therefore, they should not be pooled, their source should be specified, and the source of EVs-MVB or membrane-derived EVs ought to be considered different, at least until proven otherwise.

## 5 Conclusion

The objective of this scoping review was to evaluate the reported evidence regarding the role of EVs in idiopathic pulmonary fibrosis (IPF) and to assess the adherence of these studies to established guidelines for EVs research.

The studies were reviewed by evaluating the following steps: 1) the nomenclature used to define EVs; 2) conformity with the MISEV 2018 guidelines; 3) the biological samples used to isolate EVs; and 4) the main conclusion of each manuscript. Even if each publication revealed important innovations of EVs as biomarkers in the fibrotic response, the evidence provided by the available pioneering studies is heterogeneous. There were significant differences, such as the type and source of EVs and the analysis of EVs content, primarily a wide array of different miRNAs in the various publications that could limit the global view and reduce information. A similar observation as reported by De Lorenzis et al. review on systemic sclerosis (38) and Bergantini L. et al. (39), which emphasizes, along with our study, the need for a homogeneous approach in all the steps for the investigation of EVs, as suggested by the guidelines, to better understand the role of EVs and the possibility of comparing the results of the different investigations.

There is a need for well-precise goals and techniques for EVs isolation, identification, and content analysis. The comparison of different specimens from BAL, cells in BAL, and tissue digestion, if available, with serum might provide robust and interesting results. Crucially, future studies should focus on linking specific miRNAs to EVs derived from clearly identified cell types, rather than analyzing heterogeneous EV populations of uncertain origin. This is best achieved through controlled *in vitro* cell culture systems, which enable precise identification of cell-specific miRNA cargoes. Additionally, improving EV surface marker characterization is essential to trace their cellular source accurately, which will be key to unraveling the specific roles of miRNAs carried by EVs in IPF pathogenesis.
